# Multivalent tumor suppressor adenomatous polyposis coli promotes Axin biomolecular condensate formation and efficient β-catenin degradation

**DOI:** 10.1038/s41598-020-74080-2

**Published:** 2020-10-15

**Authors:** Tie-Mei Li, Jing Ren, Dylan Husmann, John P. Coan, Or Gozani, Katrin F. Chua

**Affiliations:** 1 Department of Medicine, Stanford University School of Medicine, Stanford, CA 94305 USA; 2 Department of Biology, Stanford University, Stanford, CA 94305 USA; 3 Medical Research Council Laboratory of Molecular Biology, Cambridge, CB2 0QH UK; 4 Education, and Clinical Center, Geriatric Research, Veterans Affairs Palo Alto Health Care System, Palo Alto, CA 94304 USA; 5 Present Address: Medical Research Council Laboratory of Molecular Biology, Cambridge, CB2 0QH UK

**Keywords:** Proteins, Biochemistry, Cancer, Molecular biology, Cell biology, Cell signalling

## Abstract

The tumor suppressor adenomatous polyposis coli (APC) is frequently mutated in colorectal cancers. APC and Axin are core components of a destruction complex that scaffolds GSK3β and CK1 to earmark β-catenin for proteosomal degradation. Disruption of APC results in pathologic stabilization of β-catenin and oncogenesis. However, the molecular mechanism by which APC promotes β-catenin degradation is unclear. Here, we find that the intrinsically disordered region (IDR) of APC, which contains multiple β-catenin and Axin interacting sites, undergoes liquid–liquid phase separation (LLPS) in vitro. Expression of the APC IDR in colorectal cells promotes Axin puncta formation and β-catenin degradation. Our results support the model that multivalent interactions between APC and Axin drives the β-catenin destruction complex to form biomolecular condensates in cells, which concentrate key components to achieve high efficient degradation of β-catenin.

## Introduction

APC mutations are present in ~ 80% of colorectal cancer cases^1^, and typically cause truncation of the APC protein. APC functions downstream of the Wnt signalosome, and it is essential for the degradation of β-catenin in the absence of Wnt stimulation^2^. APC forms a complex, termed the “β-catenin destruction complex” or “Axin degradasome” composed of β-catenin, the scaffold protein Axin, and two kinases: GSK3β and casein kinase 1 (CK1)^3,4^. In the complex, proximity of β-catenin to the two kinases leads to β-catenin phosphorylation, which in turn facilitates its ubiquitination and proteosomal degradation. Both APC and Axin are key for the function of the β-catenin destruction complex^3^. Axin is able to interact with all components of the complex: β-catenin, GSK3β, CK1, APC and Axin itself, and is believed to be the major scaffold. APC harbors multiple interaction sites for β-catenin and Axin, and an oligomerization domain^4^. Although APC is absolutely essential for the assembly of the destruction complex^5^, it’s mechanistic role is not completely understood.

The β-catenin destruction complex forms micrometer-scale punctate structures in cells^6,7^. Moreover, the punctate structures are membraneless and have high protein dynamics as determined by fluorescence recovery after photobleaching (FRAP) experiments^6–8^. Recent studies have revealed that many membraneless organelles are formed and maintained by LLPS^9–11^. This has led to a hypothesis that the punctate structures in Wnt signaling pathways are biomolecular condensates formed by LLPS^12^, an idea that is yet to be tested directly in vitro.

**Figure 1:**
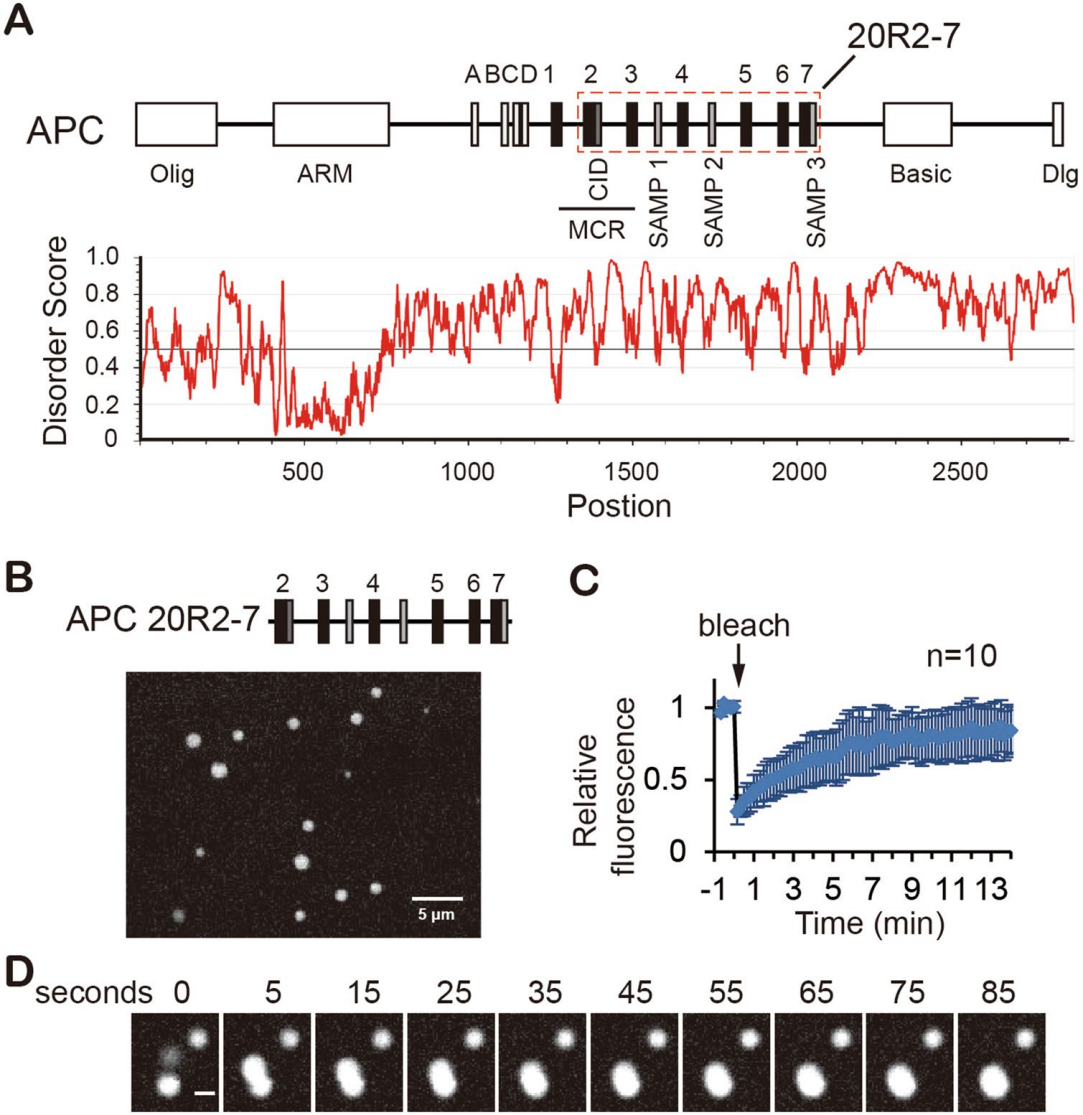
APC IDR containing multiple 20Rs and SAMPs undergoes liquid–liquid phase separation (LLPS). (**A**) Upper panel, major domains and motifs of APC protein. A-D and 1–7, 15R and 20R β-catenin interacting sites respectively; SAMP1-3, Axin interacting sites; CID, catenin inhibitory domain; MCR, mutation cluster region. Lower panel, intrinsically disorder regions of APC predicted by IUPred2A. (**B**) A representative image of the spherical structures formed by 25 µM APC 20R2-7 in 150 mM NaCl. Scale bar = 5 µm. (**C**) FRAP experiment showing the dynamics of proteins within the 20R2-7 droplet. Quantification was done with ImageJ. n = 10. Error bars are standard deviations. The average *t*_*1/2*_ is 2.71 min. (**D**) A fusion event between two liquid droplets of 20R2-7 captured by time lapse experiment. Scale bar = 2 µm.

Many proteins with LLPS properties contain IDRs comprising multiple protein–protein interacting motifs^13–15^. APC has a long IDR that contains binding sites for β-catenin (15- and 20-amino-acid repeats, i.e. 15Rs and 20Rs) and Axin (serine-alanine-methionine-proline containing sequences, i.e. SAMPs)^16,17^ (see Fig. 1A). Structural studies found that each of the 15R, 20R or SAMP motifs is able to interact with its binding partners independently^18–20^. Thus, the necessity of multiple 15R, 20R and SAMP motifs in the APC protein remains mysterious. Corroborating the importance of multiple motifs, mutations found in colorectal cancer patients often cause loss of most 20Rs and all SAMPs. Here we show that the IDR of APC that contains multiple 20R and SAMP motifs undergoes LLPS in vitro in a manner dependent on length. In the colorectal cancer cell line SW480, APC IDR promotes Axin puncta formation and efficient β-catenin degradation. Together, our data support a model in which APC IDR and its multivalent interactions with Axin promote biocondensates formation, acting to recruit and concentrate key components within a physically separated compartment and thereby increase the efficiency of β-catenin degradation.

## Results

### The APC IDR containing multiple 20R and SAMP motifs undergoes LLPS in vitro

Most APC mutations found in colorectal cancer patients are clustered in the middle of the protein, termed mutation cluster region (MCR, Fig. 1A), resulting in deletion of the C-terminal half of the protein^1^. We thus focused on the region that contain six 20Rs and three SAMPs (20R2-7) immediately downstream of the MCR, which is mostly disordered (Fig. 1A). Considering the presence of three SAMPs (Axin interacting sites) in this region, our initial attempt included characterizing both APC 20R2-7 and Axin proteins in vitro. Full-length Axin1 purified from *E. coli* and Sf9 cells didn’t yield enough protein for analysis. We observed more than one peak containing Axin1 in Size-exclusion chromatography (SEC), probably due to the polymerization of the protein through its DIX domain^21^. Precipitation was found in multiple steps of the purification process (data not shown), resulting a low protein concentration in solution that prevent us from testing its LLPS ability. In contrast, we obtained enough APC 20R2-7 protein in buffers containing 300 mM NaCl or higher. Reducing NaCl concentration results in the formation of micrometer-sized spherical granules in the solution (Fig. 1B).

To examine if the observed granules were liquid droplets, we performed time lapse imaging and fluorescence recovery after photobleaching (FRAP) experiments. SNAP-tagged 20R2-7 labeled with SNAP-Surface 549 fluorophore was spiked into the non-tagged 20R2-7 to aid in visualization of the protein. FRAP results showed that the fluorescence signal recovered within minutes after bleaching, indicating the movement of the molecules was dynamic within the droplets (Fig. 1C and Supplementary video 1–2). Time lapse experiments showed frequent and active fusion of the droplets (Fig. 1D and Supplementary video 3–4). The droplets disappeared at a NaCl concentration of 300 mM or higher, consistent with droplet formation being a reversible process. After several hours the droplets gradually matured to a solid-like state as well as formed aggregates, a process that was accelerated by the addition of the crowding reagent polyethylene glycol (PEG). Together, these results showed that the APC 20R2-7 fragment undergoes LLPS in vitro.

## LLPS propensity of APC IDR is length dependent

**Figure 2:**
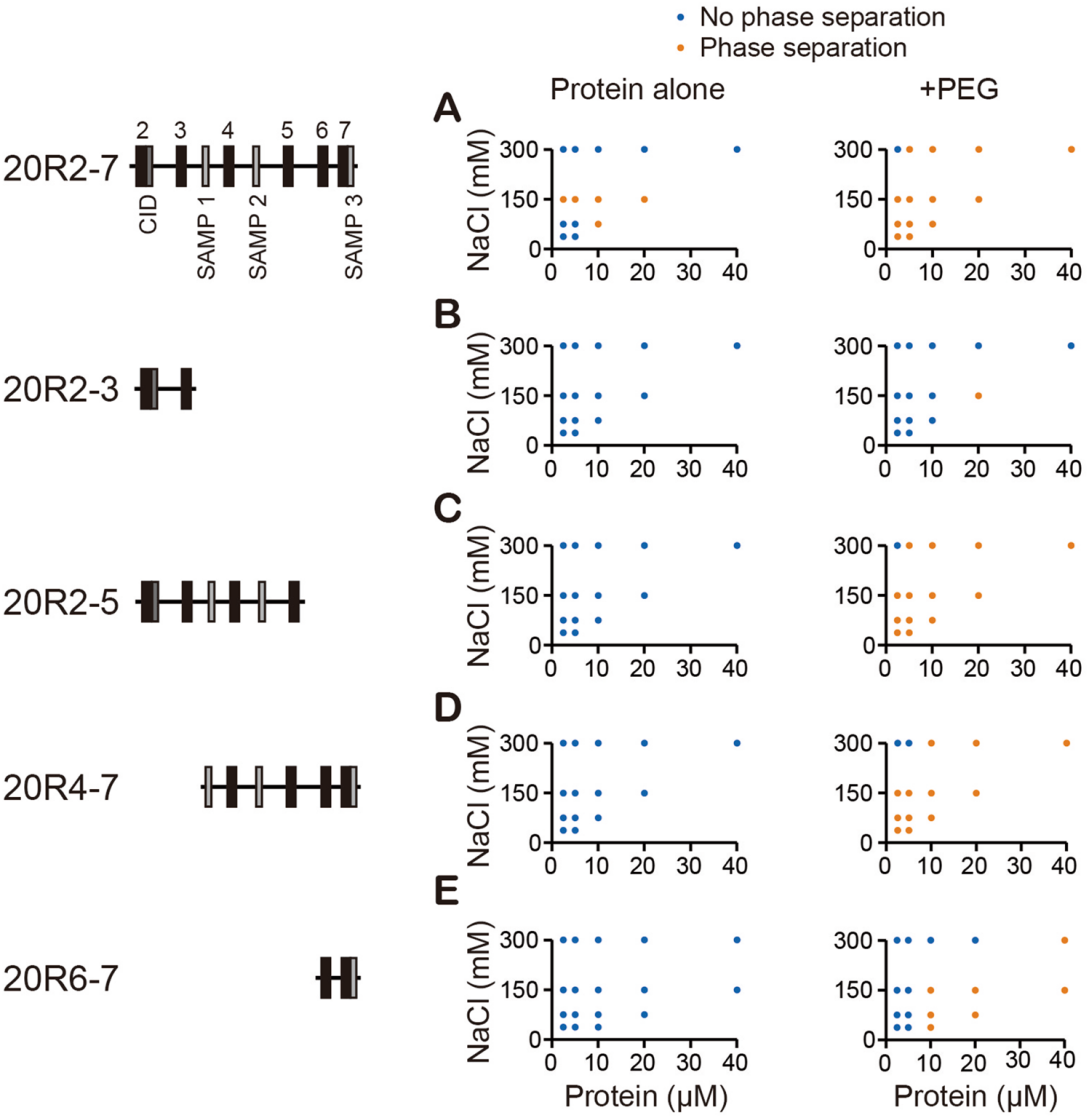
LLPS propensity of APC IDR is length dependent. Left, the APC fragments tested. (**A**–**E**) Salt diagram of the APC fragments in the absence (left panel) or present (right panel) of the crowding reagent PEG. Protein concentrations are 2.5, 5, 10, 20, 40 µM, and NaCl concentrations are 37.5, 75, 150, 300 mM.

The APC 20R2-7 consists of multiple functionally important elements, so we asked if any of them were essential for LLPS. We generated four additional recombinant APC fragments of shorter length than 20R2-7 (Fig. 2, Supplementary Fig. S1). Proteins were incubated in a range of salt (NaCl) concentrations and their propensities to form liquid droplets in vitro were assessed. Among the conditions tested, the four shorter fragments do not form liquid droplets in contrast to 20R2-7 (Fig. 2A–E left panel). The crowding reagent PEG lowered the critical concentration of 20R2-7 LLPS (Fig. 2A). We thus tested the other fragments in the presence of PEG and found that LLPS propensity correlates roughly with the length of the fragment (Fig. 2A–E right panel).

### Multivalent APC IDRs promote Axin puncta formation in cells

Next, we asked if the APC IDRs could undergo LLPS and form punctate structures in cells. We overexpressed the fragments fused to a monomeric EGFP tag (mEGFP, A206K)^22^ in SW480 cells, a cell line derived from colorectal cancer sample that only expressed a truncated APC 1-1338 aa^23^. The fragments 20R2-3, 20R2-5 and 20R2-7 all localized in the cytoplasm (Supplementary Fig. S2), likely due to a previously identified nuclear export signal (NES) near the third 20R motif^24,25^. The other two fragments 20R4-7 and 20R6-7, which lack this motif, were more uniformly distributed (Supplementary Fig. S2). Despite the seemingly regulated subcellular localization of some of the fragments, we did not observe obvious GFP puncta in these cells under current resolution with light microscopy (Supplementary Fig. S2). We reasoned that the APC IDRs are not sufficient to initiate phase separation in cells, or they may undergo some LLPS but the condensate size is limited and cannot be detected by light microscopy.

**Figure 3:**
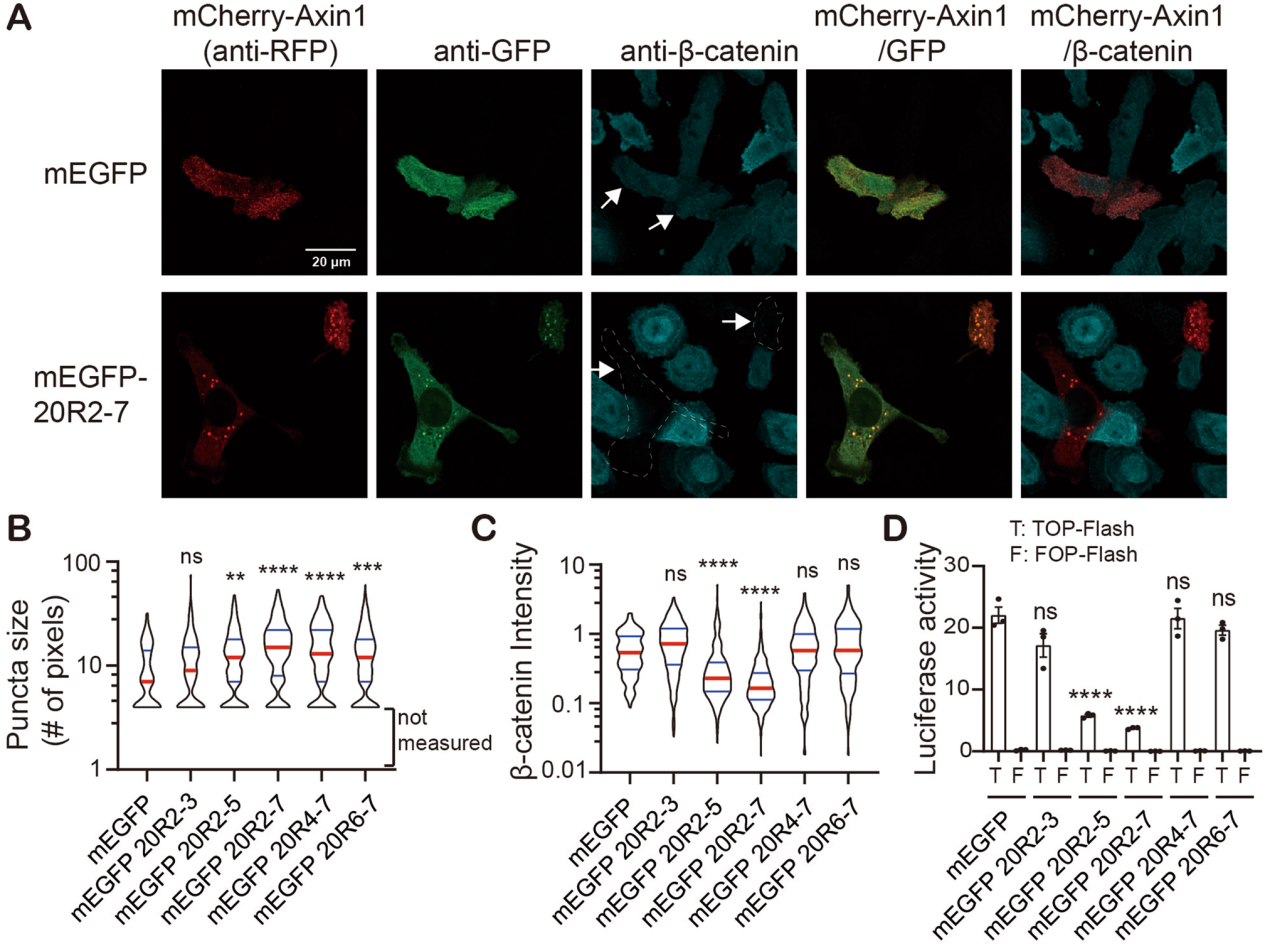
Multivalent APC IDRs promote Axin puncta formation; reduce β-catenin protein level and trans-activation activity in SW480 cells. (**A**) Immunostaining showing mCherry-Axin1 co-transfected with mEGFP or mEGFP-APC 20R2-7 in SW480 cells. Endogenous β-catenin protein level was visualized using a β-catenin antibody. Arrows indicate transfected cells. Scale bar = 20 µm. (**B**) Quantification of the size of mCherry-Axin1 puncta with diameters equal or higher than 0.5 µm. Quantification was done with CellProfiler. Puncta with smaller size were not measured due to limited resolution. Cell number > 150 for each sample. Red bar indicates median. Blue bars indicate 25% and 75% percentiles. Statistics was done with Kruskal–Wallis one-way analysis. All comparisons were made against the mEGFP control using Dunn’s multiple comparison test. ns, not significant; **p < 0.005; ***p < 0.001; ****p < 0.0001. (**C**) Quantification of β-catenin intensity in transfected cells relative to non-transfected cells. Statistics was done in the same manner as in (**B**). (**D**) TOP-Flash luciferase assay result showing the transactivation activity of β-catenin. T, TOP-Flash; F, FOP-Flash, a control with mutated TCF binding sites. One-way ANOVA analysis was used for statistics. Bar plots are mean ± SEM. ns, not significant; ****p < 0.0001.

Previous studies have suggested that Axin is the rate-limiting factor for β-catenin destruction^26,27^. Overexpression of Axin forms punctate structures in cells in a concentration dependent manner^6–8,28^. We asked if elevated Axin protein concentration cooperates with APC IDR to promote LLPS in cells. To this end, we overexpressed human Axin1 together with APC IDRs in SW480 cells. Consistent with previous observations^5^, mCherry-Axin1 forms puncta in a fraction of cells 18 h after transfection. In cells co-transfected with mEGFP-APC 20R2-7, the Axin1 puncta became more prominent relative to the mEGFP control cells (Fig. 3A). APC 20R2-7 colocalizes with Axin1 in the puncta (Fig. 3A). The shorter APC fragments increased Axin1 puncta size greater than the control but to a lesser extent than 20R2-7 (Supplementary Fig. S3A). Quantitation of the size of the puncta with diameters equal or greater than 0.5 µm demonstrated that the APC IDRs increased Axin1 puncta size in a manner that is length dependent (Fig. 3B) and which correlates roughly with their LLPS propensity in vitro (Fig. 2). Note that the fragments containing more SAMP motifs were more competent in promoting Axin puncta size comparing with their counterparts with similar length (e.g. 20R4-7 vs 20R2-5 and 20R6-7 vs 20R2-3), highlighting the importance of SAMP motifs and the interaction between APC and Axin in increasing puncta size in cells.

### Multivalent APC IDRs promote β-catenin destruction in cells

To determine the downstream consequences of APC IDR and Axin1 co-expression, we examined the level of endogenous β-catenin protein. mCherry-Axin1 expression caused a moderate reduction of β-catenin protein level compared with non-transfected cells (Fig. 3A). Co-expressing mEGFP-APC 20R2-5 or 20R2-7 with mCherry-Axin1 dramatically reduced β-catenin levels (Fig. 3A,C and Supplementary Fig. S3A). Luciferase reporter assay detecting β-catenin-dependent activation of the TCF transcription factor^29^ revealed that co-expression of mEGFP-20R2-5 or 20R2-7 with mCherry-Axin1 decreased β-catenin trans-activation activity compared to the mEGFP control (Fig. 3D). Interestingly, although 20R4-7 increased Axin puncta size (Fig. 3B), it did not further reduce β-catenin level or trans-activation activity (Fig. 3C,D). A likely explanation for this is the absence of the conserved catenin inhibitory domain (CID, also called sequence B), an essential domain for β-catenin destruction—possibly by promoting β-catenin ubiquitination, located between the second and third 20R^4,30,31^. However, the 20R2-3 fragment, which has the CID motif but has relatively short length and no SAMP motif, did not promote LLPS or reduce β-catenin level (Figs. 2, 3B–D). This suggests that the LLPS propensity of APC IDR and its CID motif are both required for β-catenin degradation.

In contrast to wildtype β-catenin, the S45A mutant, which lacks the CK1 phosphorylation site and prevents the subsequent ubiquitination and degradation^32^ accumulated in the Axin1 and APC 20R2-7 puncta (Supplementary Fig. S4), supporting the model that β-catenin is recruited to the puncta by Axin1 and APC 20R2-7.

### Multivalent APC IDRs decrease the critical concentration of Axin that required for puncta formation

**Figure 4:**
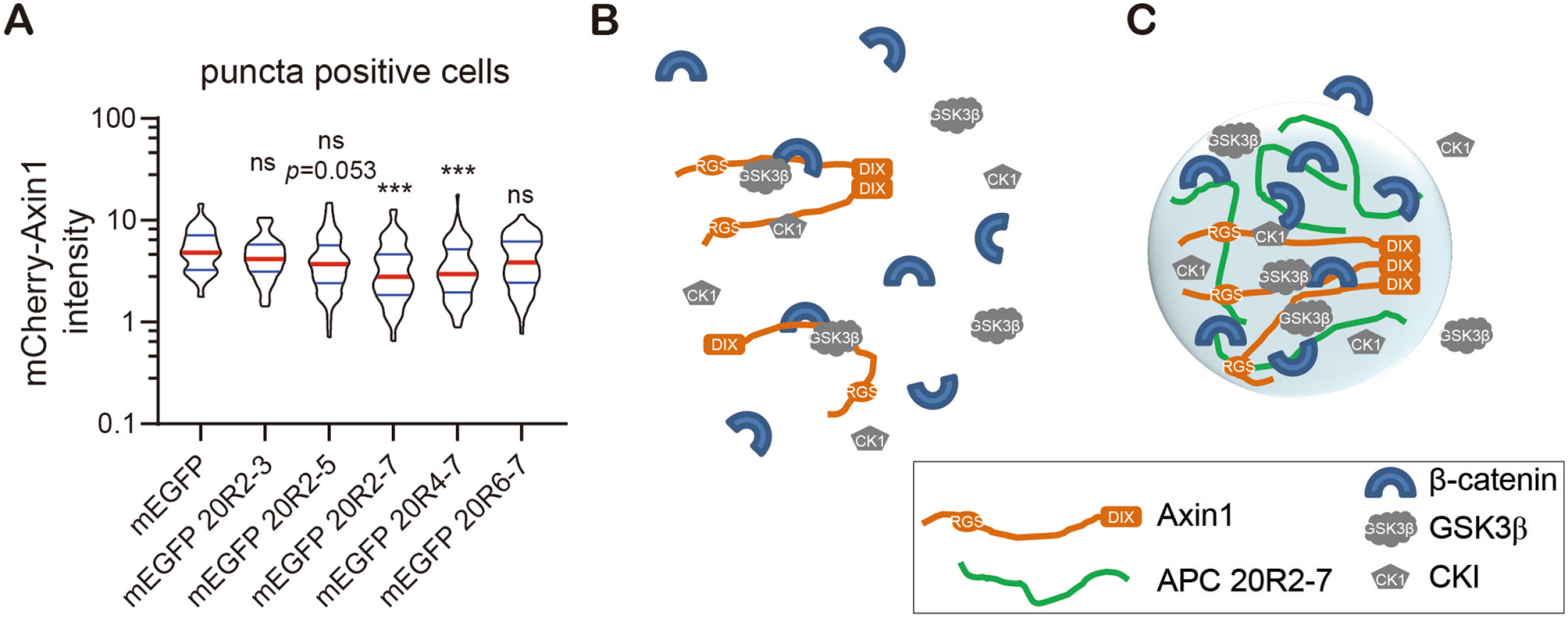
Multivalent APC IDRs decrease the critical concentration of Axin1 for puncta formation. (**A**) Quantification of mCherry-Axin1 protein intensity in puncta positive cells as identified in Fig. 3B. Red bar indicates median. Blue bars indicate 25% and 75% percentiles. Cell number > 150 for each sample. Statistics was done with Kruskal–Wallis one-way analysis. All comparisons were made against the mEGFP control using Dunn’s multiple comparison test. ns, not significant; ***p < 0.001. (**B**–**C**) Models of the diffused or punctate β-catenin destruction complex in two cells expressing similar level of mCherry-Axin1 in the absence (**B**) or presence (**C**) of APC 20R2-7. In (**B**), the DIX domain of Axin1 may undergo reversible polymerization, however it is not able to form micrometer-sized puncta due to limited concentration and/or valency of scaffold proteins. In SW480 cells, the endogenous APC (not shown) is truncated, and could not scaffold the complex efficiently. In (**C**), multivalent APC 20R2-7 provides additional valency on top of the DIX polymerization, allows puncta to form at an Axin1 concentration that it normally wouldn’t form puncta (as shown in (**B**)). Note that the puncta or the biocondensate shown in (**C**) creates a separated compartment that has extremely high protein concentration while maintaining protein dynamics.

As Axin puncta formation depends on its concentration^6–8,28^, we asked if co-expressing APC IDR altered the critical concentration of Axin that required for puncta formation. The puncta positive cells (cells with puncta that have a size equal or bigger than 0.5 µm as identified above) are those with highest mCherry-Axin1 intensity in the population (Fig. S3B) consistent with previous observations that higher Axin1 concentration promotes puncta formation^6–8,28^. We noticed that an increasing number of cells that have medium mCherry-Axin1 intensity were also puncta positive in APC 20R2-7 co-expressing cells. The quantification results confirmed this observation (Fig. 4A). Fragments 20R2-7 and 20R4-7, both have three SAMP motifs are the most potent ones. 20R2-5, which has two SAMP repeats, shows the same trend but with much smaller change compared with control (Fig. 4A). This result indicates that multivalent APC IDRs decrease the critical concentration of Axin1 required for puncta formation in cells by its multivalent interactions with Axin1.

Our results suggest that both APC IDRs and Axin1 function as scaffolds to promote the phase transition of β-catenin destruction complex. In the absence of multivalent APC IDR, Axin1 protein needs to reach a relatively high concentration in order to form micrometer-size punctate structures (biocondensates) in cells. APC IDRs containing multiple SAMP motifs increased the valency of the complex, allowing Axin1 to form micrometer-scale biocondensates at a concentration where it wouldn’t form in the absence of APC IDR (Fig. 4B,C).

## Discussion

The biocondensate model of β-catenin destruction complex has been proposed recently^12,33^. The biocondensates create physically separated compartments in the cytosol that is specifically enriched of the components of the complex. The high concentrated proteins and the dynamic nature within the compartment could allow phosphorylation and ubiquitination of β-catenin to be carried out efficiently. Moreover, the physically separated compartment ensures the specificity of enzymes such as kinases GSK3β and CK1 within the condensate. Its dynamic nature also allows rapid assembly and disassembly, which permits the precise temporal regulation by upstream signaling events.

The β-catenin destruction complex is assembled by multivalent interactions among the components^3^. Here we provide evidence supporting the model that the complex is formed by LLPS via multivalent interactions between the scaffold APC and Axin. We showed that multivalent APC IDRs are able to phase separate in vitro (Figs. 1, 2). However, they don’t form micrometer-scale macroscopic puncta when Axin protein is limited in cells (Fig. S2). Although there is a lack of direct evidence, we reason that Axin protein probably also undergoes LLPS, as it contains long disordered region and a previous study has found the polymerization of the DIX domain can be reversible^21^, two features that potentially related with phase separation. In cells, Axin promotes puncta formation in a concentration dependent manner^6–8,28^. Co-expressing multivalent APC IDRs increase the puncta size and decrease the critical concentration of Axin required for puncta formation (Figs. 3, 4), supporting the essential role of both scaffolds in promoting phase transition. This also underlines the importance of multivalency in the scaffold proteins, and is consistent with the principle that the concentration needed for phase transition is directly related to the valency of the scaffold proteins^34^. The endogenous Axin and APC proteins, although limited by their concentrations, have been observed to form very small puncta^6^, suggesting the phase transition can happen under physiological conditions. In order to understand the regulation of the complex, it will be important to examine more cell types, developmental stages and pathology conditions for the presence of the endogenous β-catenin destruction condensate.

Despite its’ scaffolding role, APC could help to control the composition of the β-catenin destruction condensate, and tune the multi-step process of β-catenin destruction. APC is a huge protein with many domains and functional motifs that interact with other proteins, creating free binding sites for low valency client proteins such as β-catenin. The free sites on the scaffold protein allows recruitment of client protein in a switch-like manner^35^, and enrich the clients into the condensate. An example is the 20R2 and CID motif that been shown to be important for the ubiquitination of β-catenin^30,31^. Our data also suggest that the CID motif is essential for β-catenin degradation and cannot be explained by simply adding valency (Fig. 3C–D and result section). It’s likely that the CID functions to concentrate a yet-to-be identified client into the condensate to promote the ubiquitination of β-catenin. In addition, truncated APC protein that lost most of the 20Rs and C-terminal sequence, as seen in many colorectal cancer patients, may assemble a condensate that have different components than the β-catenin destruction condensate^36^, which could contribute to the pathology.

The physical properties of APC might be important for the dynamics of the condensate. Interestingly, the APC 20R2-7 is highly negative charged (pI = 5.45). It has been shown that some proteins drive phase transition through heavily negative charged amino acids and their coacervation with binding partners^37^. Further analysis will need to determine if the negatively charged amino acids in APC play important roles in driving phase transition. In addition, the 20R motifs can be phosphorylated, which causes a dramatic increase in its affinity for β-catenin^19,38,39^. It would be interesting to test if and how the phosphorylation affects the phase separation propensity of APC, and how it contributes to the dynamic property of the β-catenin destruction condensate.

## Materials and methods

### Constructs

APC fragments cloned: 20R2-3 (1351–1552 aa); 20R2-5 (1351–1913 aa); 20R2-7 (1351–2070 aa); 20R4-7 (1553–2070 aa); 20R6-7 (1914–2070 aa). For *E.coli* expression, all fragments were inserted into pGEX6-P-1 vector (GE Healthcare). For fluorescence labeling of the proteins, a SNAP tag was added at the N-terminal of each fragment. For mammalian cell expression, mEGFP (monomer GFP with A206K mutation) or mEGFP-APC fragments were inserted into pLenti CMV Hygro DEST (w117-1) vector (Addgene plasmid # 17,454, a gift from Eric Campeau & Paul Kaufman)^40^. Axin-1 full-length cDNA is N-terminal mCherry tagged and inserted into pLenti6.2/V5-DEST vector (Invitrogen). The C-terminal V5 tagged in the vector was disabled by adding a stop codon after Axin-1 cDNA. β-catenin WT or S45A mutant was cloned into pcDNA3.1 vector (Invitrogen) for mammalian expression. M50 Super 8 × TOPFlash and M51 Super 8 × FOPFlash were gifts from Randall Moon (Addgene plasmid # 12,456 and 12,457)^41^. pRL-SV40P was a gift from Ron Prywes (Addgene plasmid # 27,163)^42^.

### Intrinsically disordered region (IDR) prediction

IDR prediction is done with IUPred2A^43^, using the default long disorder prediction parameters.

### Recombinant protein expression and purification

GST (Glutathione S-transferase)-tagged recombinant proteins (GST-APC fragments and GST-SNAP-APC fragments) were expressed in *E. coli* BL21 strain at 16 °C for 20 h in the presence of 0.1 mM IPTG (Sigma), purified with glutathione sepharose (GE Healthcare) in protein lysis buffer (50 mM Tris–HCl pH 7.5, 300 mM NaCl, 0.1% Nonidet P-40, 100 µg/mL RNase, 0.5 mM PMSF). GST tag was removed by PreScission enzyme in cleavage buffer (50 mM Tris–HCl pH 7.5, 300 mM NaCl, 1 mM DTT). The proteins were further purified by Size-exclusion chromatography on an AKTA-FPLC (Amersham BioSciences) equipped with a Superose 6 Increase 10/300 GL column (GE Healthcare). Fractions were checked with SDS-PAGE and coommassie blue staining. Fractions containing the protein were collected, concentrated to a final concentration of 100 µM, snap-froze in liquid nitrogen and store at -80 °C for future use. For SNAP-tagged proteins, the SNAP tag was labeled before storing with SNAP-Surface 549 nm (New England Biolabs) according to manufacturer’s manual.

### Salt diagram

Proteins stored at − 80 °C were thawed on ice. An aliquot were taken to observe under a 60 × objective lens with an EVOS M5000 Imaging System (Thermo Fisher) using bright field with differential interference contrast (DIC). Only proceed when no aggregates or particles were found in the solution. To make proteins with different concentration in various NaCl concentrations, dilute the 100 uM stock protein with dilution buffer (50 mM Tris–HCL pH 7.5, 1 mM DTT containing 0–300 mM NaCl) in a PCR tube to achieve the desired protein (2.5, 5, 10, 20, 40 µM) and NaCl concentration (37.5, 75, 150, 300 mM). The mixture was incubated at room temperature (around 25 °C) for 1 h before transferring to a µ-Slide 8 Well Glass Bottom Chamber (Ibidi) and observed with the same microscope and objective. Granules were determined by their signature Brownian movement in the solution before settling down to the bottom of the chamber.

### Time lapse and FRAP

Time lapse and FRAP experiments were carried out with a 3i spinning disk confocal microscope equipped with a 63 × objective lens (CSU-W1 SoRa). Protein APC 20R2-7 was prepared in the same way as salt diagram experiment, except that 5% of SNAP-549 nm labeled 20R2-7 was added to facilitate visualization. 20R2-7 protein concentration was 25 µM and NaCl concentration was 150 mM. The experiments were carried out between 30 and 60 min after making the sample. Image was taken every 5 (time lapse only) or 10 s (FRAP), 100 frames total. For FRAP experiment, each bleach area was equal or less than 50% of the total area of the selected droplet. FRAP images were quantified with NIH ImageJ software^44,45^ (version 1.50i) and intensity corrected and normalized as described below. To eliminate noise signal, background intensity was subtracted for all droplets in the frame. The intensity of each bleached area was then normalized to unbleached droplets in the same frame to remove the global photobleaching during image acquisition. The intensity of each bleached area was compared with the average intensity of pre-bleach (5 images) and plotted. The half time *t*_1/2_ was calculated based on a non-linear regression curve fit. Average *t*_1/2_ of 10 movies was used.

### Cell culture and transfection

SW480 cells (ATCC) was cultured in Dulbecco’s modified Eagle’s Medium (DMEM, Life Technologies) supplemented with 10% fetal bovine serum and penicillin–streptomycin (Life Technologies) at 37 °C incubator with 5% CO_2_.

For transfection, cells were plated on 0.1% gelatin (EMD Millipore) coated cover glasses (Warner Instruments, CS-12R 12 mm) in a 24 well cell culture plate (Thermo Fisher). Transfection was done using lipofectamine 3000 reagent (Thermo Fisher) following manufacturer’s manual. Amount of plasmids used for each well of a 24 well plate: mEGFP or mEGFP-APC fragment, 150 ng; mCherry-Axin, 50 ng; FLAG-β-catenin, 150 ng.

### Immunostaining

18 h after transfection, cells were fixed with 4% paraformaldehyde, washed and blocked with 2% BSA in PBS containing 0.3% Triton X-100 (PBST). Cells were then incubated with primary antibodies at 4 °C overnight, washed with PBS and incubated with secondary antibodies in PBST for 1 h at room temperature. Nuclear was stained with 0.5 µg/mL DAPI in PBST (Sigma) for 10 min and washed with PBS twice. The cover glasses were then removed from the plate and mounted on microscope slides (Fisher) with Fluoro-Gel (Electron Microscopy Sciences).

Primary antibodies used: chicken-anti-GFP (Aves Labs Inc. # GFP-1020, 1:2000), rabbit-anti-RFP (Rockland # 600-401-379, 1:500), mouse-anti-β-catenin (BD Bioscience # 610153, 1:500), mouse-anti-FLAG M2 (Sigma # F1804, 1:50). Secondary antibodies used (1:500 dilution): donkey-anti-rabbit conjugated with Cy3 (Jackson ImmunoResearch # 711-165-152, 1:500); donkey-anti-chicken conjugated with Alexa Fluor 488 (Jackson ImmunoResearch #703-545-155); Goat-Anti-Mouse conjugated with Alexa Fluor 647 (Jackson ImmunoResearch 115-605-003).

### Confocal microscopy and quantification

Images of cells were taken on confocal microscopes, a Zeiss 780 confocal microscope or a 3i spinning disk confocal microscope (CSU-W1 SoRa). 40 × objective lens were used. For quantification, areas on each slide contain at least 150 transfected cells and 1000 non-transfected cells were imaged using the 3i spinning disk confocal microscope. Quantification of cell intensity and puncta size were done with CellProfiler (version 3.1.8)^46^. Due to limit of resolution, only puncta with a diameter equal or greater than 0.5 µm were identified and their size measured. mCherry-Axin intensity and β-catenin intensity were normalized to the endogenous β-catenin intensity in non-transfected cells (average intensity of over 1000 cells) on each slide before comparing across slides. Statistics and Plots were done with GraphPad Prism. The data did not pass a normal distribution test. Thus non-parametric one-way ANOVA (Kruskal–Wallis one-way analysis) test and Dunn’s multiple comparisons with correction were used.

### Luciferase assay

mCherry-Axin and/or mEGFP-APC fragment were cotransfected with luciferase reporter plasmids into SW480 cells. Three replicates were performed for each condition in a 96 well plate. Amount of plasmid used for each well: mCherry-Axin (10 ng); mEGFP-APC fragment (30 ng); M50 TOPFlash or M51 FOPFlash (20 ng); pRL-SV40P renilla luciferase reporter (20 ng) as a control for transfection. 18 h after transfection, cells were lysed, and firefly and renilla luciferase substrates added sequentailly according to the manual of Dual-Glo Luciferase Assay System (Promega). Luciferase emission was measured using a microplate reader (Biotek, synergy H1). Firefly luciferase activity of each well was normalized to the renilla luciferase activity of the same well before comparing between different conditions. Three independent biological replicates were done.

### Western blotting

Cells were lysed with RIPA buffer and protein concentration determined by Bradford assay. The same amount of total proteins across samples was denatured by adding 5X SDS sample buffer (250 mM Tris–HCl pH = 6.8, 10% SDS, 30% glycerol, 5% β-mercaptoethanol, 0.02% bromophenol blue)and boiled at 100 °C for 15 min. 10 µg of total protein was loaded on a SDS-PAGE and blotted with anti-FLAG M2 (Sigma F1804) or anti-β-tubulin antibody (Sigma 05-661). Secondary antibody used was peroxidase conjugated goat-anti-mouse (Jackson Immunoresearch #715–035-151).

## Supplementary information

Supplementary Information.

Supplementary Video 1.

Supplementary Video 2.

Supplementary Video 3.

Supplementary Video 4.
