# Supplementary material for: Multivalent tumor suppressor adenomatous polyposis coli promotes Axin biomolecular condensate formation and efficient β-catenin degradation
Source: Sci Rep. 2020 Oct 15;10:17425. doi: 10.1038/s41598-020-74080-2 (PMC7562749; doi:10.1038/s41598-020-74080-2)

Supplementary Information

Multivalent Tumor Suppressor Adenomatous Polyposis Coli Promotes Axin Biomolecular Condensate Formation and Efficient β-catenin Degradation

Tie-Mei Li[1,2ǂ*], Jing Ren[3], Dylan Husmann[2], John P. Coan[1,2], Or Gozani[2*], Katrin F. Chua[1,4*]

[1]Department of Medicine, Stanford University School of Medicine, Stanford, CA 94305, USA
[2]Department of Biology, Stanford University, Stanford, CA 94305, USA
[3]Medical Research Council Laboratory of Molecular Biology, Cambridge CB2 0QH, United Kingdom
[4]Geriatric Research, Education, and Clinical Center, Veterans Affairs Palo Alto Health Care System, Palo Alto, CA 94304, USA
[ǂ]Current address: Medical Research Council Laboratory of Molecular Biology, Cambridge CB2 0QH, United Kingdom

*corresponding authors: Tie-Mei Li: tiemei.li2@gmail.com, Or Gozani: ogozani@stanford.edu, and Katrin F. Chua: kfchua@stanford.edu

Supplementary Figure S1. Coomassie staining of APC fragments after removing of GST tag, showing the relative size of the fragments.

Supplementary Figure S2. APC fragments do not form micrometer-sized puncta when overexpressed in SW480 cells. Confocal microscope images showing SW480 cells transfected with mEGFP tagged APC IDRs or mEGFP alone as control. Nuclei are stained with DAPI. Scale bar = 20 μm.

Supplementary Figure S3. (A) Representative immunofluorescence images of SW480 cells co-transfected with mCherry-Axin1 and mEGFP tagged APC 20R2-3, 20R2-5, 20R4-7 or 20R6-7. Endogenous β-catenin protein level was visualized using a β-catenin antibody. Arrows indicate transfected cells. Scale bar = 20 μm. Quantification results are shown in Figure 3B-C. (B) Quantification of mCherry-Axin1 intensity in all transfected cells (left, n > 1,000 cells for each sample) or puncta positive cells (right, also see Fig. 4A). Red bar indicates median. Blue bars indicate 25% and 75% percentiles.

Supplementryl Figure S4. The β-catenin S45A mutant accumulates in the Axin and APC puncta. (A) FLAG tagged β-catenin S45A was co-transfected with mCherry-Axin1 and mEGFP or mEGFP-APC 20R2-7. An anti-FLAG antibody was used to visualize the FLAG-β-catenin protein. (B) Western blotting showing the total protein level of FLAG-β-catenin WT and S45A in different transfected cells. Two full blots loaded with the same amount of samples were shown.

Supplementary video 1. A representative FRAP experiment of an APC 20R2-7 droplet. Scale bar = 2 μm.

Supplementary video 2. A representative FRAP experiment of an APC 20R2-7 droplet. Scale bar = 2 μm.

Supplementary video 3. A representative fusion event between two droplets formed by APC 20R2-7. Scale bar = 2 μm.

Supplementary video 4. A representative fusion event between two droplets formed by APC 20R2-7. Scale bar = 2 μm.

Li_Supp Fig S1

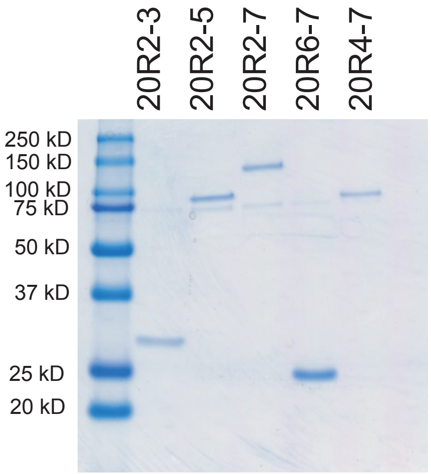

Li_Supp Fig S2

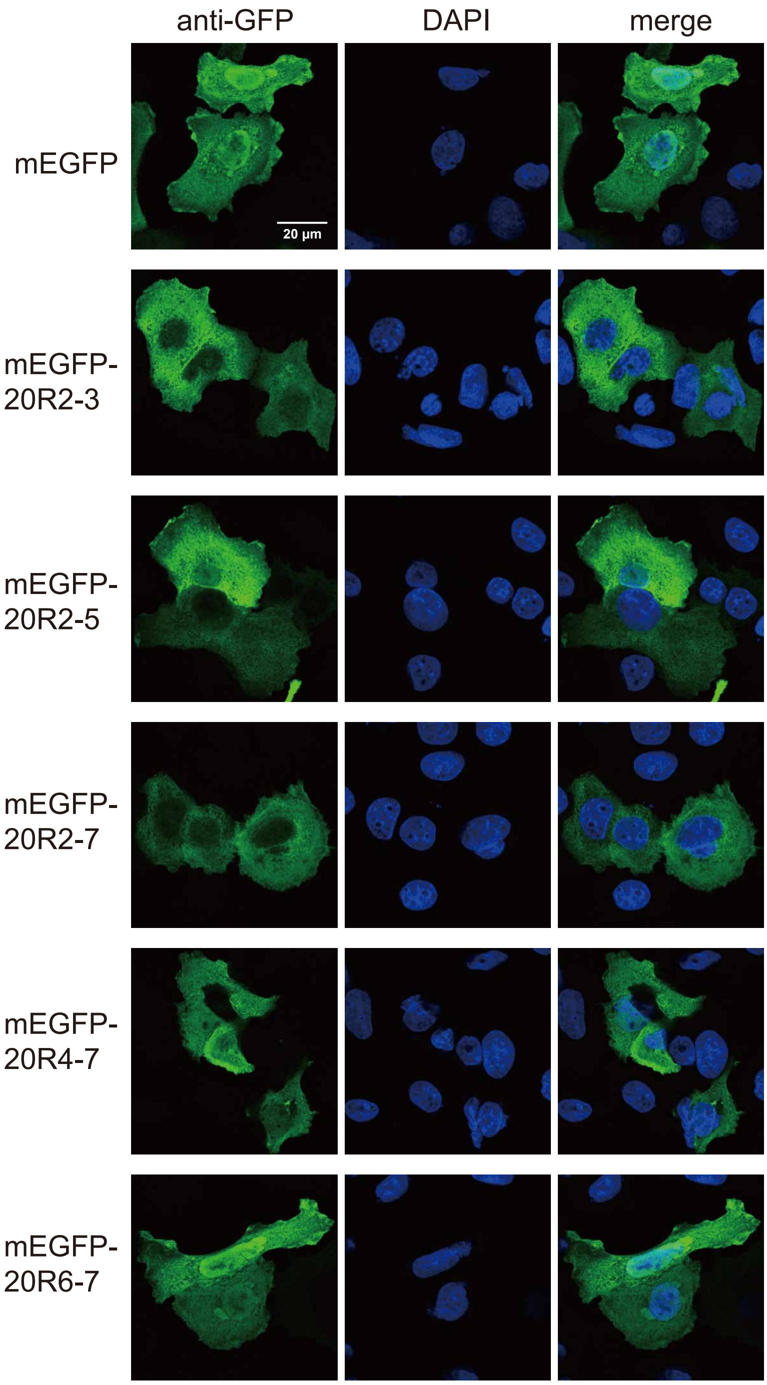

Li_Supp Fig S3

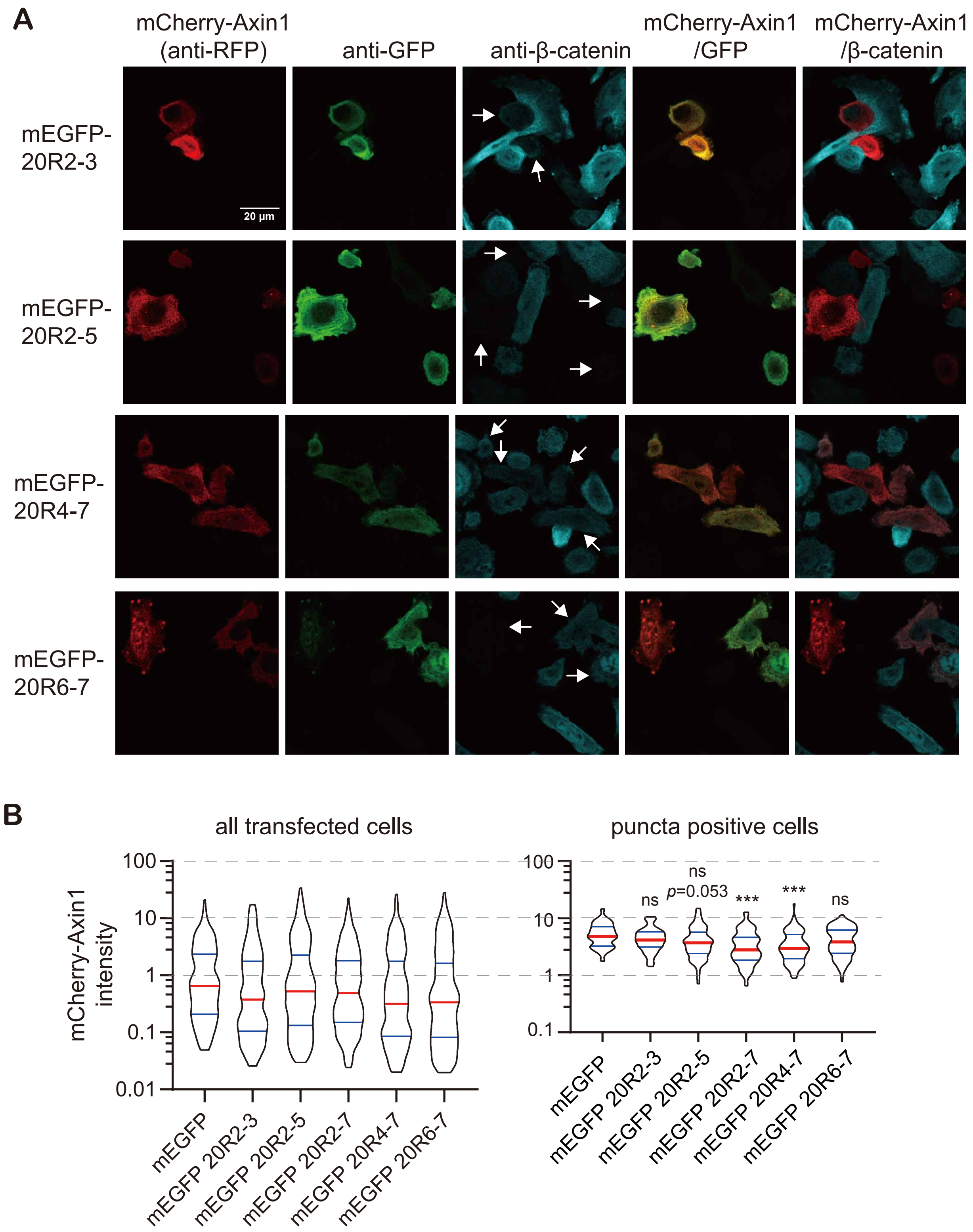

Li_Supp Fig S4

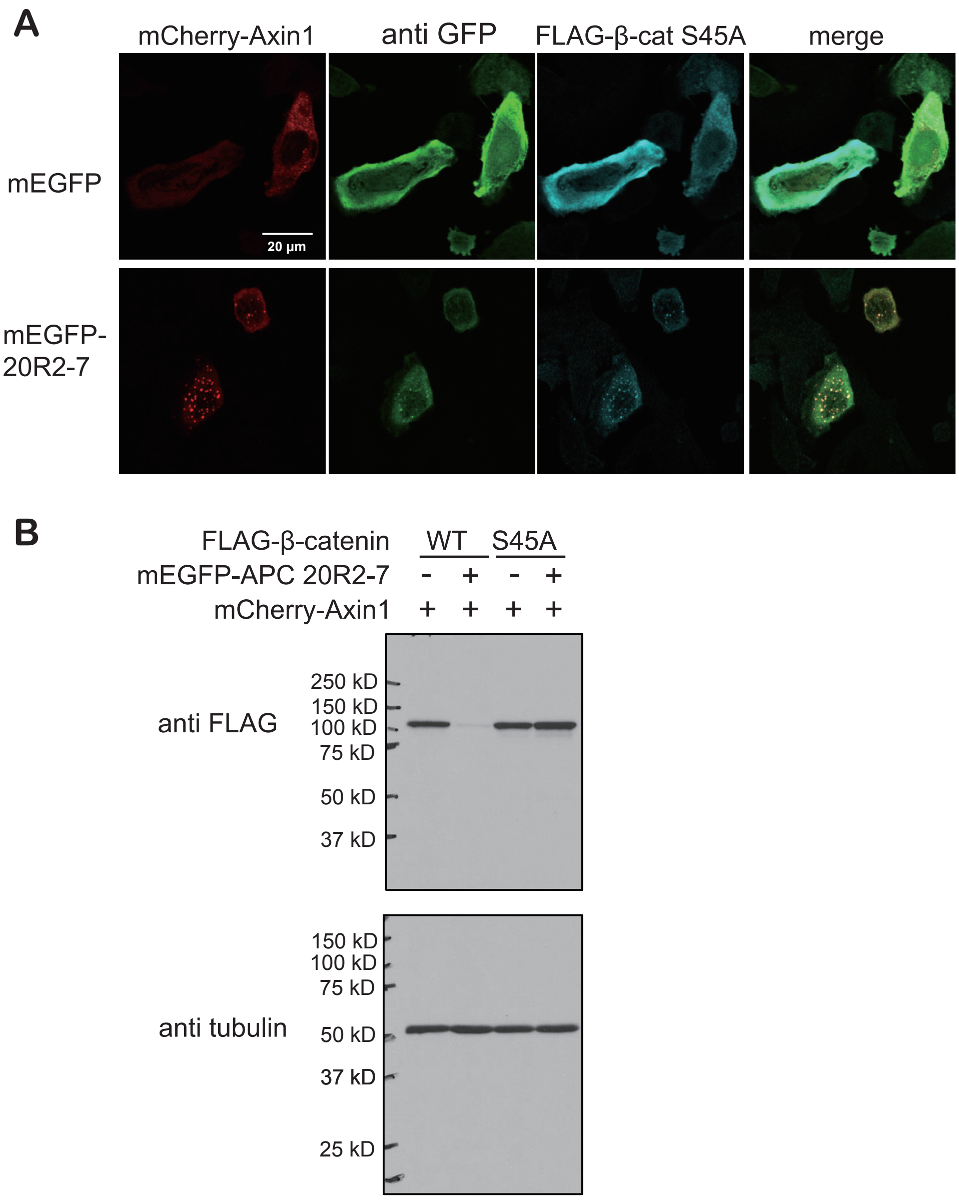

Supplement: Supplementary file 1 — Supplementary Information. [file 41598_2020_74080_MOESM1_ESM.pdf]
